# Innate immune cells in vascular lesions: mechanism and significance of diversified immune regulation

**DOI:** 10.1080/07853890.2025.2453826

**Published:** 2025-01-23

**Authors:** Jinjing Wu, Yulu Qian, Kuang Yang, Shuhua Zhang, Erming Zeng, Daya Luo

**Affiliations:** a School of Basic Medical Sciences, Jiangxi Medical College, Nanchang University, Nanchang, Jiangxi, China; b Queen Mary University of London, Nanchang University, Nanchang, China; c Jiangxi Provincial People’s Hospital, The First Affiliated Hospital of Nanchang Medical College, Jiangxi Cardiovascular Research Institute, Nanchang, Jiangxi, China; d Department of Neurosurgery, The First Affiliated Hospital, Jiangxi Medical College, Nanchang University, Nanchang, Jiangxi, China

**Keywords:** Innate immune cells, angiogenesis, vascular lesion, immune regulation, immunotherapy

## Abstract

Angiogenesis is a complex physiological process. In recent years, the immune regulation of angiogenesis has received increasing attention, and innate immune cells, which are centred on macrophages, are thought to play important roles in vascular neogenesis and development. Various innate immune cells can act on the vasculature through a variety of mechanisms, with commonalities as well as differences and synergistic effects, which are crucial for the progression of vascular lesions. In recent years, monotherapy with antiangiogenic drugs has encountered therapeutic bottlenecks because of the short-term effect of ‘vascular normalization’. The combination treatment of antiangiogenic therapy and immunotherapy breaks the traditional treatment pattern. While it has a remarkable curative effect and survival benefits, it also faces many challenges. This review focuses on innate immune cells and mainly introduces the regulatory mechanisms of monocytes, macrophages, natural killer (NK) cells, dendritic cells (DCs) and neutrophils in vascular lesions. The purpose of this paper was to elucidate the underlying mechanisms of angiogenesis and development and the current research status of innate immune cells in regulating vascular lesions in different states. This review provides a theoretical basis for addressing aberrant angiogenesis in disease processes or finding new antiangiogenic immune targets in inflammation and tumor.

## 1. Introduction

Angiogenesis is the process of forming, growing, and remodelling new blood vessels from a preexisting vascular system, which is essential for transporting blood to tissues and organs throughout the body to meet the oxygen requirements and provide various nutrients needed for the body’s activities as well as for transporting metabolic end products to excretory organs. The formation, development, and maturation of the vascular system is a complex process consisting of three main stages: (1) the formation and proliferation of vascular sprouts, (2) the extension and anastomosis of vascular sprouts and initial formation of vascular lumens to the interflow of blood, and (3) remodelling of the vascular network and pericyte coverage to the point of vascular maturation [1]. This process often depends on the balance of pro- and antiangiogenic factors. Immune cells are capable of producing and releasing large amounts of proangiogenic mediators in response to different stimuli, such as vascular injury, hypoxia, tumours, trauma, and inflammatory responses, which are involved in endothelial cell proliferation, migration, and activation. Therefore, immune regulation during angiogenesis becomes extremely important.

In immunology, innate immune cells are divided into three categories: classical innate immune cells, innate lymphoid cells (ILCs) and innate-like lymphocytes (ILLs) [2], which constitute the first line of defence against foreign pathogens. In recent years, immunotherapy with innate immune cells at the core has gradually become a research hotspot. Targeting immune cells to reshape the tumor microenvironment has provided a new ideas for tumor immunotherapy. Inherent immune cells exist in the blood and most organs of the body and have close interactions with the vascular system. On the one hand, endothelial cells mediate the recruitment of immune cells to extravascular tissues by expressing leukocyte adhesion molecules. On the other hand, soluble factors secreted by innate immune cells affect the behaviour of endothelial cells and regulate vascular maturity and stability [3].

In the steady state, the angiogenesis and remodelling of blood vessels are in a state of balance. When the balance is disturbed by internal and external stimuli, blood vessels will be overactivated or degenerated, thus forming a chaotic vascular network, such as excessive vascularization in tumours, which provides an opportunity for tumour metastasis and growth [4]. In fact, this pathological vascular change also widely occurs in many cardiovascular diseases and has become an important factor affecting the occurrence, development and prognosis of diseases, thus, antiangiogenic therapy has good prospects. While antiangiogenic treatment can improve treatment outcomes, the overall benefit for patient survival is still rather limited. This makes drug resistance to antiangiogenic therapy a major challenge. Tumor cells can not only undergo non-angiogenic vascularization through vascular mimicry and vascular co-option, but also resist antiangiogenic therapy through metabolic symbiosis, perivascular invasion, the upregulation of angiogenic factors [5] and autophagy [6]. The regulation of vascular stability and maturation by tumor stromal cells such as endothelial cells, pericytes, adipocytes and infiltrating immune cells is also one of the mechanisms of antiangiogenic therapy resistance [7]. In addition, the substitution of the vascular endothelial growth factor (VEGF) signaling pathway for the angiogenesis pathway [8] and the hypoxia microenvironment [9] caused by antiangiogenic drugs can also induce angiogenesis and vascular remodelling, thus triggering drug resistance effect. An increasing number of studies have shown that antiangiogenic drugs alone or in combination with chemotherapy or other targeted therapies have a limited impact on the overall survival rate of cancer patients and rarely result in durable responses [10].

At present, antiangiogenic therapy combined with immunotherapy, especially immune checkpoint inhibitor therapy, has significantly improved the therapeutic effect on various solid tumours and has played an increasingly important role in clinical application [11]. The strategy of ‘vascular-immune cross-linking’ involves a positive feedback loop with synergistic effects. On the one hand, antiangiogenic drugs create a short ‘vascular normalization’ window, promote the infiltration and activation of immune cells, and provide favorable conditions for immunotherapy [12]. On the other hand, when immune cells are activated, they will secrete a large amount of IFN-γ, which can effectively promote the normalization of tumor blood vessels [13]. Therefore, finding immune targets that cooperate with antiangiogenic therapy may provide potential combined strategies for treating vascular diseases. An in-depth understanding of the dynamic changes of immune cells in the vascular microenvironment in normal and disease states is expected to surpass traditional treatment protocols for vascular-related diseases and achieve better efficacy through novel combination therapy methods.

## 2. Molecular mechanisms of angiogenesis

The development and maturation of new blood vessels is a precise, complex and multistage coordinated process, that includes the activation of endothelial cells (ECs), the selection and migration of tip cells, the proliferation and extension of stalk cells, vascular bud anastomosis and vascular remodelling. The sprouting of blood vessels is the basis of angiogenesis. When blood vessels sprout, the cells at the forefront of the branches are called tip cells, and the cells next to the tip cells are called stalk cells. The differentiation of tip/stalk cells depends on Dll4-Notch signalling and is guided by vascular endothelial growth factor receptor (VEGFR) [14,15]. Tip cells activated by VEGF can emit radial filamentous pseudopodia, which are used to guide the direction of blood vessel germination and control angiogenesis and simultaneously secrete metalloproteinases to promote the migration of ECs by decomposing the basement membrane area [16]. Stalk cells mainly prolong the branches of new blood vessels. When stalk cells proliferate, the lumen is gradually extended until the tip cells from two adjacent arteries meet and fuse, and finally, a new vascular circuit is established [17]. Research has shown that the process of vascular anastomosis is also subject to immune regulation. Macrophages have has good mobility and flexibility in this process, and have been shown to have high affinity for filamentous pseudopodia of tip cells, which can guide the fusion of tip cells and promote the formation of a vascular network [18]. The integration and tightening of ECs adjacent to new blood vessels need to be specifically connected by cell adhesion molecules such as VE cadherin [19]. VE cadherin is a key component of endothelial adhesion molecule connections (AJs), which play an important role in maintaining vascular integrity and promoting vascular stability.

**Figure 1.**
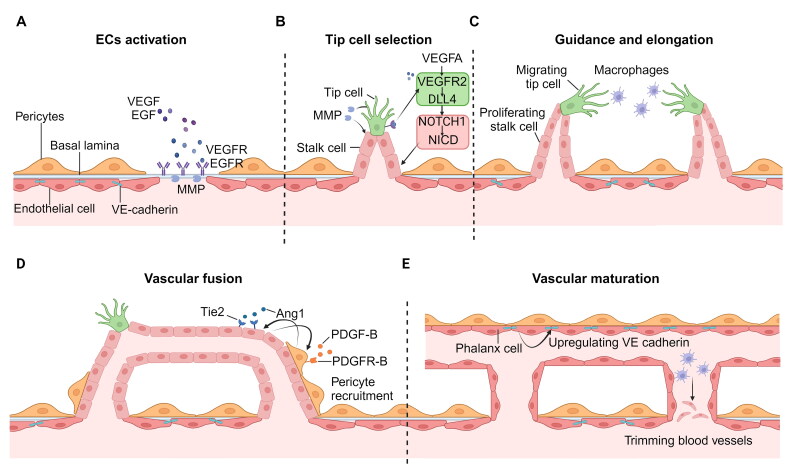
**The process of neovascularization and vascular maturation.** (a) Matrix metalloproteinases (MMP) degrades the basement membrane and extracellular matrix, and the exposed growth factor receptor is stimulated by angiogenic growth factor to promote ECs activation. (b) The selection of tip and stalk cells is regulated by Dll4-Notch signalling and VEGFR2 expression levels. (c) The proliferation of stalk cells, the migration of tip cells and the guidance of macrophages induce the anastomosis of two germinated blood vessels. (d) Recruiting pericytes to maintain the stability of blood vessels after the anastomosis of new blood vessel buds. (e) ECs become static and nonproliferating phalanx cells and form stable vascular cavities under the trimming of macrophages, the covering of peripheral cells and the connection of cadherin. (created with BioRender.com).

After adhesion and anastomosis between blood vessels, blood perfusion begins in the new blood vessels, and the stable endothelial cells, which are called phalanx cells, enter a static state without proliferating. This type of cell forms cobblestone-like monolayer cells, which promote vascular stability by upregulating VE cadherin [17,20] The remodelling and maturation of new blood vessels are also closely regulated by mural cells (MCs). The tip cells, which are the first to induce vascular germination, recruit nearby peripheral cells through platelet-derived growth factor (PDGF)B signalling [21] and use it as an attractant to induce the migration of peripheral cells near the vascular endothelium [22]. Angiopoietin (Ang) has also been indicated to be necessary for vascular wall cells to stabilize blood vessels. Ang1 can activate Tie2 receptors on endothelial cells, induce Tie2 phosphorylation and then lead to AKT activation, thus promoting the survival pathway in ECs, inhibiting the apoptosis pathway and maintaining the stability of blood vessels [23,24]. After the formation of a stable vascular lumen, the smaller vascular network may fuse and lead to vascular dilation, degenerate or be swallowed up and pruned by immune cells, and result in the formation of an orderly and regular vascular network [25] (Figure 1).

## 3. Regulation of innate immune cells in vascular homeostasis

Under normal conditions, adult vascular endothelial cells are usually quiescent, but within the female reproductive system, angiogenesis is a fundamental process in the cyclic regeneration of the endometrium, embryo implantation, and placental development. Among these, angiogenesis is very active at the maternal-fetal interface [26]. To date, angiogenesis in the reproductive tract is regulated by at least 20 pro-angiogenic and inhibitory factors. Of these, VEGF shows cell-specific expression and is the most critical regulator [27]. In addition, the regulation of angiogenesis is an important part of the wound healing process. The wound healing process consists of four phases: the hemostatic phase, the inflammatory phase, the proliferative phase and the remodeling phase. Regulation of angiogenesis occurs mainly in the latter two phases [28].

Innate immune cells can dynamically support and orchestrate the process of physiologic angiogenesis. Under homeostatic conditions, monocytes can differentiate into non-classical patrol monocytes or tissue-resident macrophages [29]. Non-classical patrolling monocytes ‘move’ and ‘crawl’ along the luminal surface of the vascular endothelium in a lymphocyte function related antigen (LFA-1) and integrin α4-dependent manner [30]. In a recent study, highly migratory cells in the chorionic allantoic membrane (CAM) of the chick embryo were observed ‘patrolling’ the capillary-forming region on day 9 of embryonic development. Migrants recruited more monocytes by releasing CXCL12, and released VEGFA to promote angiogenesis. The researchers identified this population of cells as monocytes by single-cell sequencing and antibody staining [31]. At steady state, LYVE1^+^ macrophages have a unique ability to regulate the arterial extracellular matrix, which maintains normal arterial elasticity by binding to hyaluronic acid (HA) expressed by smooth muscle cells (SMCs) and mediating the MMP9-dependent process of collagen degradation [32]. A study has shown that macrophages can perform specific functions to restore tissue integrity at different stages of skin repair after mechanical injury. Early in the repair response, macrophages are responsible for controlling granulation tissue formation and myofibroblast differentiation. In the middle stage of the repair response, macrophages are mainly responsible for stabilizing vascular structures and inducing the transition from granulation tissue to scar tissue [33]. More interestingly, it was found that during the process of tissue repair, macrophages could also promote endothelial cell articulation at both ends of damaged blood vessels through direct physical adhesion and mechanical traction to achieve rapid repair of the broken endothelium [34]. Physiologically, the regulation of angiogenesis by NK cells and dendritic cells occurs mainly at the maternal-fetal interface where the vascular network is abundant. NK cells at the maternal-fetal interface are mainly uterine NK (uNK) cells. uNK cells participate in angiogenesis and vascular remodeling in normal placental development. They are not only closely related to the vascularization of decidua and the formation of spiral arteries in human and mice, but also can synthesize some cytokines and vasoactive mediators related to vascular stability [35]. Similarly, DCs in the vicinity of uterine vessels are directly involved in the regulation of decidual angiogenesis through the production of VEGF, soluble vascular growth factor receptor Flt-1 (sFlt-1), and transforming growth factor-β (TGF-β) 1 [36,37]. In summary, innate immune cells are able to dynamically support and orchestrate the process of physiological angiogenesis. They promote vascular stability and maintain vascular physiological functions mainly by secreting VEGF or regulating ECs.

## 4. Regulation of innate immune cells in vascular lesions

In 1996, the International Society for the Study of Vascular Abnormalities (ISSVA) divided vascular diseases into proliferative vascular diseases (called ‘vascular tumours’) and relatively static vascular malformations [38], which are often congenital. The former is characterized by excessive proliferation of ECs, whereas the latter has no abnormal proliferation of vECs, which involves abnormal expansion and communication of blood vessels with normal endothelial cell tissue structure and biological characteristics [39]. This abnormal proliferation is harmful as it mainly leads to vascular rupture and bleeding or vascular embolism. When the body is in a state of disease or inflammation, the pathological changes in the blood vessels themselves are different because of the different lesion sites and disease types.

There are still two kinds of pathological patterns of blood vessels in the disease state: relatively dynamic vascular proliferative changes and relatively static vascular obstructive changes. Vascular proliferative lesions usually comprise the abnormal proliferation of endothelial cells, accompanied by functional defects or morphological abnormalities in perivascular cells such as pericytes [40] and the continuous destruction of VE cadherin, which plays a connecting role [41]. The resulting vascular network is often uncontrolled and disorderly. Immature blood vessels lacking the combination of mural cells can easily lead to excessive penetration, poor perfusion and increased hypoxia. This abnormal vascularization is common in tumours. Vascular obstructive diseases often involve abnormalities in the internal tissue structure of the vascular wall, including EC injury, proliferation and hypertrophy of vascular smooth muscle cells (vSMCs), and an imbalance in extracellular matrix (ECM) composition, which eventually leads to lumen stenosis and occlusion [42,43]. Most of these changes are caused by vascular wall thickening induced by vascular wall sclerosis and vascular wall inflammation, which may lead to ischaemia of donor tissues or even necrosis of tissues, organs and limbs and are common in diseases such as atherosclerosis, thromboangiitis obliterans and Takayasu’s arteritis [44,45]. Here, we mainly discuss the influence of innate immune cells on vascular diseases such as tumours and atherosclerosis (AS).

### 4.1. The role of innate immune cells in vascular proliferative lesions

In the process of tumour development, tumour cells need oxygen and nutrients to survive and proliferate, so these cells need to be close to blood vessels to enter the blood circulation system. Early observation revealed that fast-growing tumours involve extensive angiogenesis, so Judah Folkman suggested that tumour progression needed to start tumour angiogenesis [46]. However, the vascular network formed with the help of high-level angiogenic factors is often abnormal, and is characterized by highly irregular vascular morphology, such as twisting, uneven diameter, branching and disordering, and highly immature vascular structures, such as loose EC connections and incomplete coverage of peripheral cells, which leads to vascular leakage [47]. VEGF is the core factor involved in tumour angiogenesis. The increase in VEGF‐A levels not only promotes the proliferation and survival of ECs but also causes the leakage and bending of new blood vessels, resulting in irregular blood flow. In addition, VEGF‐A has been proven to play a key role in increasing the invasiveness, vascular density, metastasis and recurrence of tumours [48]. Several other growth factors, including Ang, PDGF-B and TGF-β family members, are also related to the formation of defective vascular networks in tumours [49].

At present, the therapeutic strategies targeting VEGF/VEGFR take the tumour vascular microenvironment as the breakthrough point, which can cut off the nutritional source of tumour cells and inhibit tumour growth. In addition, it can correct the abnormal structure and function of tumour blood vessels and improve the antitumour effects [50]. However, antiangiogenic agents may excessively prune tumour vessels in a dose- and time-dependent manner, and excessive elimination of the tumour vasculature may increase the hypoxia and immunosuppression in the tumour area and increase the difficulty of drug delivery, thus leading to the failure of patient treatment [10]. Currently, an increasing number of animal models and clinical trials have validated the role and effectiveness of immunotherapy combined with antiangiogenic therapies. In the tumour microenvironment (TME), the abnormal angiogenesis in tumour is caused by many factors, and many studies have revealed the complex regulatory mechanism of innate immune cells in abnormal angiogenesis. Innate immune cells may provide potential immunotherapeutic targets for increasing the efficacy of antiangiogenic therapy and producing synergistic antitumor effects. The key regulatory mechanisms of innate immune cells on blood vessels in the TME are summarized in Table 1.

**Table 1.** Mechanisms of vascular immune regulation in tumor and atherosclerosis.

Cell type	Tumour	Atherosclerosis
Monocytes	TEMs: Endothelial cells upregulate Ang-2 to recruit TEMs to the tumour site. TEMs promotes angiogenesis by secreting a series of angiogenic factors [52]. M-MDSCs: (1) Secrete a series of angiogenic factors. (2) Transdifferentiate into endothelial cells. (3) The expression of high level MMPs further stimulates angiogenesis and tumour growth [53,54].	M1-like macrophages secrete inflammatory cytokines that lead to endothelial cell dysfunction and activate proliferation of vSMCs [98]. (2) M2-like macrophages near blood vessels in advanced plaques are able to exert anti-inflammatory effects and promote restoration of blood flow [99]. (3) vSMCs- monocyte/macrophage interactions: macrophages promote the proliferation and migration of SMCs [100,101]. SMCs contribute to monocyte adhesion and recruitment [102]. SMCs transformed into macrophage-like phenotype [103]. cSMCs maintain the reparative phenotype of macrophages [104].
Macrophages	TAM: (1) Secrete various angiogenic factors, chemokines, chemokine receptors and protease [58,59,61]. (3) Directly interaction with ECs [62]. (4) Metabolic regulation of TAMs [64]. (5) M1-like macrophages inhibit angiogenesis and promote vascular regression by inducing endothelial-to-mesenchymal transition [63].	
NK cells	TANK/TINK: (1) Produce high-level angiogenic factors [68,69]. (2) Interacting with other immune cells such as mast cells and neutrophils [70,71]. (3) Tumour cells release immuno- suppressive soluble factors, regulating the recruitment of NK cells and the transformation of pro-angiogenic phenotype [67].	(1) Release perforin and granzyme B to promote the progression of AS and the expansion of necrotic core [107]. (2) Influence AS progression through inflammatory responses, oxidative stress and apoptotic effects [108]. (3) NKT cells influence plaque stability through interactions with vSMCs [(109].
DCs	pDC/imDC: (1) Release angiogenic factors or antiangiogenic factors to directly act on ECs [72–76]. (2) Release cytokines to affect the reactivity of ECs to vascular growth factors [81,82]. (3) Release cytokines to promote other types of cells to produce angiogenic factors or antiangiogenic factors [77]. (4) Release extracellular matrix (ECM) components that can interfere with angiogenic factors [78[. (5) DCs trans- differentiates into endothelial-like cells [80].	(1) Damaged endothelium enhances DCs adhesion and migration [111]. (2) Antigen presentation or production of inflammatory cytokines [112,113]. (3) pDC secretes IFN-α to promote apoptosis in vSMCs [114]. (4) CCL17^+^ DC accelerates atherosclerosis by limiting Tregs [113].
Neutrophils	TAN: (1) Release MMP9 to promote tumour angiogenesis [84]. (2) Secrete elastase to promote the expression of VEGF in tumour cells [86]. (3) Produce anti-angiogenic factors [89,90].	(1) Release myeloperoxidase (MPO), protease, adhesion molecule and nitric oxide [118,119]. (2) Promote inflammatory responses in AS through NETs [120]. (3) Neutrophil-Monocyte/Macrophage Interactions [116, (121]. (4) Neutrophil-EC interactions [122,123].

#### 4.1.1. Monocytes

**Figure 2.**
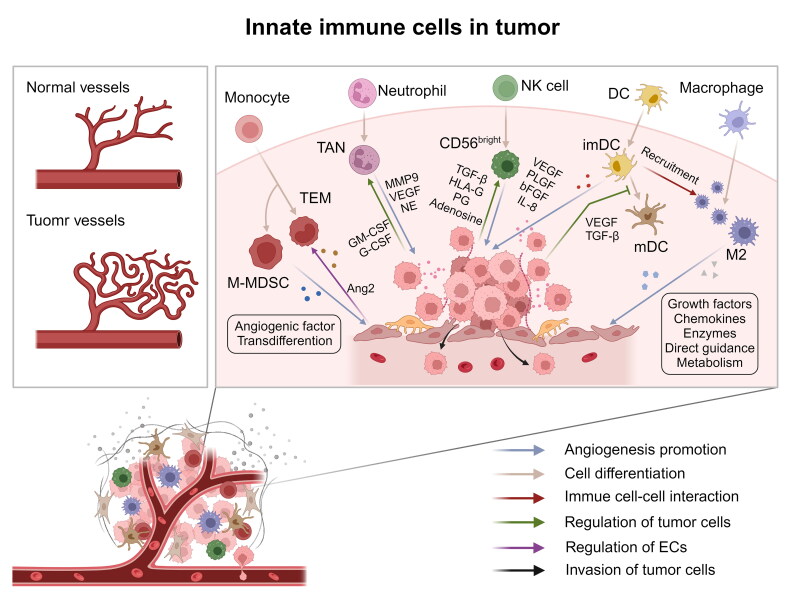
**The role of innate immune cells in tumour angiogenesis.** In the TME, the secretion of high-level angiogenic factors promotes highly irregular tumour blood vessels, such as distorted shapes, different diameters and disordered branches. Loose EC connections and incomplete coverage of peripheral cells lead to vascular leakage. Monocytes in the TME participate in tumour angiogenesis through transdifferentiation and the secretion of angiogenic factors. Neutrophils exert angiogenic activity by secreting NE, VEGF and MMP9. Moreover, tumour cells also regulate the angiogenic ability of neutrophils by producing GM-CSF and G-CSF. TAMs regulate angiogenesis through paracrine and direct effects on endothelial cells and lead to vascular overactivation through metabolic regulation. ImDCs and decidual CD56^bright^ NK cells can produce high levels of angiogenic factors, such as VEGF, PLGF, bFGF and IL-8. ImDCs secrete cytokines to promote the aggregation of inflammatory cells such as macrophages under the action of TGF-β. Cytokines such as VEGF and TGF-β secreted by tumour cells can also inhibit the maturation of infiltrating DCs and release immunosuppressive soluble factors such as TGF-β, HLA-G, prostaglandin and adenosine to regulate the recruitment and phenotypic transformation of NK cells. ECs can also react to immune cells, for example, ECs can recruit TEMs to tumour sites by upregulating Ang-2. (created with BioRender.com).

A unique lineage of proangiogenic monocytes, namely, Tie2-expressing monocytes (TEMs), has been found in the peripheral blood and tumours of cancer patients. TEMs in the tumour microenvironment have excellent angiogenic activity. TEM knockout completely prevents the formation of glioma neovascularization in the mouse brain and induces substantial tumour regression [51]. Endothelial cells recruit TEMs to tumour sites by upregulating Ang-2, and TEMs exert their angiogenic activity by secreting a series of angiogenic factors, such as VEGF, MMP9, COX2 and Wnt5A [52]. Monocyte myelogenous inhibitory cells (M-MDSCs), which accumulate in the TME, were also found to have angiogenic characteristics. On the one hand, M-MDSCs mediate angiogenic activity by expressing VEGF, bFGF, PDGF and MMP9. On the other hand, the expression of endothelial markers such as VEGFR and VE cadherin in tumour is significantly increased, which promotes angiogenesis and tumour growth through direct differentiation and integration into the vascular endothelium [53,54] (Figure 2). Monocytes mainly participate in the regulation of tumour blood vessels through transdifferentiation and paracrine signalling.

#### 4.1.2. Macrophages

Vascular homeostasis is regulated by many proangiogenic and antiangiogenic factors. When these two types of factors are in equilibrium, the vascular system is at rest, and ECs are in a nonproliferative state. When the proangiogenic signal is dominant, it induces the start of angiogenesis, which is called the ‘angiogenesis switch’ in tumours [55]. In the TME, tumour-associated macrophages (TAMs), which indirectly regulate the ‘angiogenesis switch’ mainly by affecting the expression of vasoactive factors and proteolytic enzymes, have been proven to be important drivers of cancer angiogenesis [56]. According to the different activation states and functions induced by the microenvironment, activated macrophages are usually divided into two categories, M1-like macrophages and M2-like macrophages [57]. M2-like macrophages play a powerful role in promoting angiogenesis in three main ways. First, they secrete cytokines to promote angiogenesis. M2-like macrophages can secrete a variety of angiogenic factors (VEGF, TGF-β, PDGF, Ang, and bFGF [58]), chemokines (CXCL2, CXCL4, and CXCL12) and chemokine receptors (CXCR3, CXCR4, CXCR8, and CXCR9 [59]) to promote angiogenesis. Among them, many angiogenic factors often play a synergistic role. For example, PDGFBB can recruit pericytes and mesenchymal stem cells. Without PDGFBB, the blood vessels formed only under the stimulation with VEGF are immature and easily leak and degenerate [60]. Second, proteases are secreted to promote angiogenesis. M2-like TAMs can also promote angiogenesis by producing different kinds of enzymes, such as MMP family members, especially MMP2 and MMP9, as well as plasmin and urokinase plasminogen activator (uPA). These proteases can destroy the stability of blood vessels, degrade the adventitia, create space for angiogenesis, and then promote angiogenesis [61] (Figure 2). The third mechanism involves direct interaction with ECs. M2-like macrophages are in direct contact with vECs and their surrounding matrix, which promotes the germination, fusion and remodelling of blood vessels. Blood vessel budding is the first step for preexisting blood vessels to form new blood vessels. The growth of blood vessel buds in tumours can stimulate tumour cells again by secreting growth factors [62]. Research on infantile haemangioma (IH) has shown that M2-like macrophages promote the angiogenesis in IH by regulating the behavior of ECs and the differentiation of stem cells during the early stage of IH proliferation. In contrast, M1-like macrophages showed anti-proliferative and antitumour effects during the late stage of IH proliferation, inhibited angiogenesis and promoted vascular regression. Phenotypic transformation of macrophages and macrophage-EC interactions may provide a new directions for alleviating the progression of IH [63].

In addition, angiogenesis is regulated by metabolism. In the hypoxic tumour microenvironment, TAMs strongly upregulate the expression of the negative regulatory factor REDD1 of mTOR, and the inhibition of mTOR mediated by REDD1 can hinder glycolysis in TAMs, causing ECs to dominate glucose competition with TAMs, leading to overactivation of the endothelium and blood vessels and thus causing abnormal vascular structure [64]. Recent studies have shown that reprogramming TAMs can inhibit tumor angiogenesis. TAMs deficient in tuberous sclerosis complex 1 (Tsc1) need more nutrients to maintain their growth. They grow near blood vessels and have sufficient nutrients, thus competing with vascular endothelial cells, which can reshape the structure of blood vessels in tumours, inhibit angiogenesis, and cause tumour cells to be far from blood vessel necrosis in a large area [65].

#### 4.1.3. NK cells

Natural killer cells are effector lymphocytes involved in tumour immune monitoring. Although the role of NK cells in tumor blood vessels has not been fully studied, it has been pointed out that NK cells in highly invasive and metastatic triple negative breast cancer are mainly immature CD11b^-^CD27^-^ NK cells, which may be related to tumor metastasis and angiogenesis [66]. In solid malignant tumours, tumour-associated NK (TANK) cells and tumour-infiltrating NK (TINK) cells in the peripheral blood usually exhibit phenotypic changes to low-cytotoxicity NK cells [67]. The main subgroup of NK cells in the normal parenchyma of lung tissue is CD56^dim^CD16^+^ cells, whereas in patients with non-small cell lung cancer (NSCLC), TANK and TINK cells can be transformed into the decidual CD56^bright^CD16^-^phenotype, and these cells can produce high levels of angiogenic factors, such as VEGF, PLGF and IL-8 [68,69]. Therefore, similar to decidual NK (dNK) cells, TINKs and TANKs have several markers of mature NK cells, including granzyme and perforin, but these TINK and TANK cells have low cytotoxicity and produce angiogenesis-promoting factors.

NK cells also interact with other immune cells, such as mast cells [70] and neutrophils [71], to regulate their existence and activation as well as cooperate to establish a proangiogenic response in the process of tumour growth and progression. In addition, the TME itself is also involved in the regulation of the functional activity of NK cells. Tumour cells release soluble immunosuppressive factors, such as TGF-β, HLA-G, prostaglandin and adenosine, which regulate the recruitment of NK cells and promote the phenotypic transformation of angiogenesis (Figure 2). Among these factors, TGF-β can polarize NK cell precursors and even mature NK cells into the CD56^bright^CD16^-^VEGF^high^PLGF^high^CXCL8^+^IFN-γ^low^ NK cell subset, which has angiogenic activity, and has been identified as the main angiogenic phenotype conversion factor of NK cells in the decidua and TME [67]. The TME and NK cells interact with each other, and the tumour cells reprogram NK cells to perform their own phenotypic functions work for themselves and promote the progression of tumours.

#### 4.1.4. DCs

DCs can express a wide range of angiogenic and antiangiogenic mediators and regulate angiogenesis through different mechanisms: (1) DCs directly act on ECs by releasing proangiogenic factors (such as VEGF-A, FGF2 and ET-1 [72,73]) or antiangiogenic factors (such as IL-18, IFN-α, CXCL9, CXCL10 and CCL21 [74–76]). (2) Cytokines released by DCs affect the responsiveness of ECs to vascular growth factors. For example, DCs can increase the expression of vascular growth factor receptors on EC surfaces [77]. (3) DCs release cytokines to promote the production of angiogenic factors or antiangiogenic factors by other types of cells. (4) The ECM released by DCs can interfere with proangiogenic factors. For example, the platelet-reactive protein 1 (TSP-1) produced by DCs is a powerful antiangiogenic molecule that can simultaneously inhibit the activities of FGF2, VEGFA and hepatocyte growth factor (HGF) [78]. The relationship between DCs and tumour angiogenesis was first discovered in an ovarian cancer study, which revealed that tumour-related plasma cell-like DCs (pDCs) induced angiogenesis *in vivo* by producing TNF-α and IL-8, whereas mDCs derived *in vitro* inhibited angiogenesis *in vivo* by producing IL-12. However, tumour ascites contain many pDCs but not mDCs. Therefore, tumour cells may increase angiogenesis by attracting pDCs while excluding mDCs to prevent angiogenesis from being inhibited, thus providing a new mechanism for regulating tumour angiogenesis [79].

In fact, the phenotype and function of DCs are often influenced by angiogenic factors present in the TME. Recent observations have shown that abundant cytokines and lactate in the TME induce monocytes to differentiate into tumour-related DCs, which transdifferentiate into endothelial-like cells in the presence of angiogenic mediators [80]. According to the literature, cytokines secreted by fast-growing tumours, such as VEGF and TGF-β, can also inhibit the maturation of infiltrating DCs. These immature dendritic cells (imDCs) around tumours will not only lead to the immune tolerance in tumour cells but also promote the formation of new blood vessels and induce ECs to colonize and differentiate. ImDCs promote the generation, development and maturation of tumour blood vessels mainly by attracting ECs in the blood circulation into the tumour and generating paracrine signals (such as VEGF, IL-8 and bFGF) to support the formation of new blood vessels [81,82]. Moreover, under the action of TGF-β, imDCs secrete cytokines to promote the aggregation of inflammatory cells such as macrophages, and the interaction of these cells may increase their ability to promote angiogenesis (Figure 2). For example, under the induction of imDCs, macrophages can promote angiogenesis by secreting IL-1, causing the infiltrated cells to secrete endothelial cell-activating factor [77]. In summary, DCs in the TME may promote the formation of new blood vessels through different mechanisms: stimulating the angiogenesis of existing blood vessels by releasing angiogenic factors, promoting angiogenesis by transforming into endothelial-like cells, and indirectly promoting angiogenesis by regulating other immune cells. In these cases, VEGF-A seems to play a key role. As a growth factor that is directly produced by DCs, VEGF-A inhibits the functional maturity of DCs and causes them to differentiate into endothelial-like cells.

#### 4.1.5. Neutrophils

In recent years, studies have shown that neutrophils have phenotypic and functional plasticity. Clinical research has shown that the number of neutrophils in the peripheral blood and tumour tissue of tumour patients increases, which is closely related to malignant progression and poor prognosis of tumours [83]. Under the action of tumours and their microenvironment, neutrophils can undergo phenotypic and functional changes, promote tumour occurrence, growth and metastasis, promote tumour angiogenesis and mediate tumour immunosuppression. Neutrophils are the source of soluble media that play an important role in angiogenesis. In cancer, MMP9 released by neutrophils can promote tumour angiogenesis [84]. In the Rip-Tag2 model of islet carcinogenesis, neutrophils expressing MMP-9 mainly exist in the angiogenic islets of dysplasia and tumours, and the short-term depletion of neutrophils significantly reduces the frequency of initial angiogenesis in dysplasia [85]. In addition, neutrophil elastase (NE) secreted by neutrophils can promote the expression of VEGF in tumour cells [86]. In the TME, neutrophils and tumour cells are regulated in two ways. For example, in invasive breast cancer, cancer cells induce tumour-associated neutrophils (TANs) to release oncostatin M (OSM) by producing granulocyte-macrophage colony stimulating factor (GM-CSF) and through cell–cell contact, which induces cancer cells to produce VEGF, promotes tumour blood vessels and increases cancer cell invasion ability [87]. Tumour-derived granulocyte colony stimulating factor (G-CSF) can also induce neutrophils to express the Bv8 protein, thus promoting local angiogenesis [88] (Figure 2). In addition, neutrophils also produce important antiangiogenic factors. For example, neutrophils can synthesize and secrete human neutrophil peptides (HNPs), also known as α-defensins. Antimicrobial peptides inhibit EC adhesion, migration and proliferation in a fibronectin-dependent manner, damage capillary formation *in vitro* and reduce angiogenesis *in vivo* [89]. Neutrophil-derived elastase can also produce the antiangiogenic factor angiostatin [90]. The latest research conducted single-cell RNA sequencing (scRNAseq) and assays for transposase-accessible chromatin followed by sequencing (ATACseq) on neutrophils from different organs and tumours through an orthotopic model of pancreatic ductal adenocarcinoma and revealed that tumour infiltrating neutrophils aggregated and developed into a persistent ‘T3’ cell subgroup. T3 neutrophils have the highest level of VEGFA expression, which promotes angiogenesis and tumour growth, highlighting that targeting the local neutrophil response still has great potential as an immunotherapy [91].

### 4.2. The role of innate immune cells in vascular obstructive diseases

AS, as a chronic inflammatory disease, is the main pathological basis of cardiovascular and cerebrovascular diseases and is characterized by chronic inflammatory reactions in the arterial wall. Endogenously modified structures are deposited on the intima of arteries, which stimulates the accumulation of immune cells, ECs and SMCs, produces proinflammatory mediators and fibrous fat plaques, and finally leads to hardening, loss of elasticity and narrowing of the arterial lumen [92]. Endothelial cell dysfunction (ECD) is considered a typical feature of early AS lesions and can be induced by factors such as decreased NO bioavailability, vascular oxidative stress, the inflammatory response and haemodynamics [93].

In the steady state, the sugar calyx on the EC surface is beneficial for regulating vascular permeability, and its thickness is greater than that of adhesion molecules expressed by ECs, which can prevent inflammatory cells from adhering to the endothelium. However, the thickness of the glycocalyx decreases in the atherosclerotic plaque area, which leads to the exposure of vascular adhesion molecules such as intercellular adhesion molecule-1 (ICAM-1) and vascular adhesion molecule-1 (VCAM-1) expressed by ECs [94], and circulating monocytes are selectively recruited from the blood into the intima and differentiated into macrophages and then transformed into foam cells due to lipid metabolism disorders. A variety of chemokines and growth factors produced by activated ECs and macrophages act on adjacent vSMCs (or their precursors), inducing their proliferation and synthesis of ECM, thus producing fibrous plaques [95]. The edges of these plaques contain abundant inflammatory cell groups (activated macrophages, T cells, NKT cells and DCs), which further affect the function of vSMCs, regulate changes in the proinflammatory phenotype of ECs, and lead to unstable plaque structure through proteolytic modification of ECM components, which exacerbates inflammation and obstruction of the vascular lumen. When an unstable plaque ruptures, the necrotic core can easily cause the release of thrombogenic substances, which can lead to AS thrombosis [93,96].

Therefore, immune cells play important roles in the occurrence and development of AS. ECs and immune cells release a variety of proinflammatory factors, activate cytokines, chemokines, bioactive lipid compounds and adhesion molecules, increase local inflammation and promote the development of AS lesions. Table 1 summarizes the key regulatory mechanisms of innate immune cells in atherosclerotic vascular lesions.

#### 4.2.1. Monocytes/macrophages

**Figure 3.**
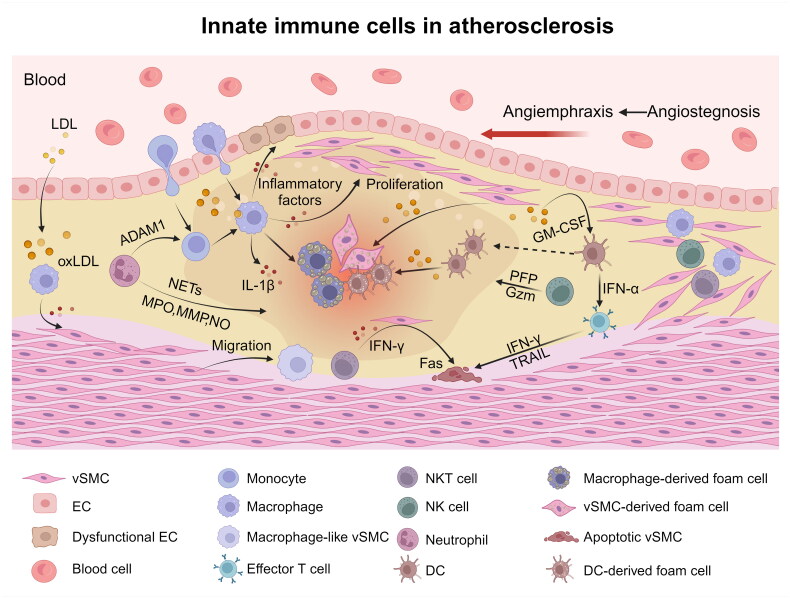
**The role of innate immune cells in atherosclerosis.** After oxidation, LDL becomes oxLDL. Macrophages in blood vessels and differentiated monocytes, DCs and vSMCs phagocytose oxLDL and transform it into foam cells, which exacerbates the expansion and necrosis of plaques. Macrophages closely interact with vSMCs, and inflammatory cytokines secreted by macrophages can cause endothelial cell dysfunction, activate the proliferation of vSMCs, and transform vSMCs into a macrophage-like phenotype. Neutrophils mainly participate in the pathological process of AS by activating degranulation, forming NETs, secreting cytokines and regulating other immune cells. NK cells and NKT cells in AS mostly gather around macrophages in plaques. The perforin and granzyme B secreted by NK cells contribute to atherosclerosis and the expansion of the necrotic core. IFN-γ secreted by NKT cells can promote the expression of Fas on the surface of vSMCs, thus causing the apoptosis of vSMCs and affecting plaque stability. OxLDL stimulates ECs to produce GM-CSF, which can increase the adhesion and migration of DCs and regulate the number of DCs in plaques. IFN-α secreted by pDCs can induce effector T cells to upregulate IFN-γ and TRAIL and then kill vSMCs in AS through a TRAIL-DR5-dependent apoptosis mechanism, which may lead to plaque instability. In short, the innate immune cells in AS affect the stability and integrity of plaques through different mechanisms, which can easily lead to plaque shedding and thrombosis, leading to blood vessel blockage. (created with BioRender.com).

Macrophages are the most important immune inflammatory cells involved in the occurrence and progression of AS, and there are two kinds of macrophages in plaques: vascular macrophages *in situ* and macrophages differentiated from monocytes. Both of them can swallow LDL cholesterol and form foam cells rich in intracellular lipid droplets, but there are some nonfoam CCR2^+^ macrophages in the latter, which can express proinflammatory factors such as IL-1β to participate in inflammatory reactions [97] (Figure 3). There are also macrophages with different phenotypes in AS. In the early stage of the lesion, M2-like macrophages are located mainly in the plaque, and the number of M1-like macrophages gradually increases with the progression of the lesion. M1-like macrophages mainly play a proinflammatory role, and secreted inflammatory cytokines can lead to endothelial cell dysfunction and activate the proliferation of vSMCs, thus promoting thrombosis and aggravating vascular occlusion [98]. M2-like macrophages near neovascularization in advanced plaques mainly play an anti-inflammatory role, which can promote the recovery of blood flow and inhibit the development of lesions to some extent [99].

The vSMC-macrophage interaction in the arterial wall also plays an important role in the formation of AS. Wang et al. reported that, in an apoE-KO mouse model, 70% of the total foam cells were derived from SMCs in mice fed a western diet for only 6 weeks, and the macrophages and SMCs were almost mixed throughout the whole thickened intima after the mice were fed a western diet for 12 weeks. However, there were no SMCs in the intima of the mice at the beginning of AS occurrence. This rapid progress may be because cytokines secreted by ECs and infiltrating macrophages induce SMCs to migrate from the middle arterial layer of mice, and SMCs quickly flood into the intima and proliferate and ingest lipids. Moreover, SMCs come into direct contact with macrophages as soon as they reach the intima [100,101]. Many *in vitro* studies have shown that the interaction between macrophages and SMCs can promote the proliferation of SMCs and the synthesis and migration of proteoglycans and matrix metalloproteinases. In contrast, SMCs also contribute to the adhesion, recruitment and survival of monocytes [102]. The interaction between SMCs and macrophages can also aggravate the inflammation of these two cell types and transform SMCs into a macrophage-like phenotype [103]. The latest research used *in vivo* imaging and single-cell transcriptome analysis in mice, and revealed that there is a special subgroup of SMCs in the atherosclerotic plaque area, which are called chemotactic SMCs (cSMCs). These cells actively maintain the repair phenotype and strategic distribution of macrophages in the vascular niche by using proinflammatory signalling molecules such as CCL2 and MIF, revealing the ‘vascular macrophage niche’ and the regulation of the maturation and distribution of these vascular macrophages [104].

#### 4.2.2. NK cells

Studies have shown that immune cells such as NK cells and NKT cells in AS appear at the lesion site of AS [105], and these two types of cells mostly gather around macrophages in plaques. NK cells only account for only 0.1%–0.5% of the total lymphocytes in AS lesions of mice and humans [106]. Nevertheless, NK cells play a very important role in the development of AS *in vivo*. Selathurai et al. reported that treating apoE^-/-^ mice with an Asialo-GM1 antibody can greatly reduce the pathological degree of AS pathology after exhausting NK cells. Researchers subsequently transferred NK cells from wild-type mice to ApoE^-/-^Rag2^-/-^IL-2rg^-/-^ mice and reported that the size of AS lesions doubled, thus confirming the role of NK cells in promoting AS. To determine whether the function of NK cells in AS depends on the production of IFN-γ or cytotoxin, researchers have compared the transplantation of NK cells lacking IFN-γ, perforin and granzyme B with that of wild-type NK cells. The results showed that the production of perforin and granzyme B contributed to the expansion of atherosclerosis and the necrotic core [107]. NK cells not only participate in the progression of AS through perforin and granzyme but also participate in inflammatory reactions, oxidative stress and apoptosis, which can jointly affect the formation of AS. Animal experiments have confirmed that the selective deletion of NK cells leads to an increase in plasma cholesterol levels, an increase in the AS lesion area, the aggravation of vascular occlusion, lipid metabolism disorders and strong inflammatory reactions in mice [108]. In addition, NKT cells in AS can also affect the stability of plaques through interactions with vSMCs. IFN-γ secreted by NKT cells can promote the expression of Fas on the surface of vSMCs, thus causing the apoptosis of vSMCs and affecting plaque stability [109] (Figure 3). Although NK cells account for a small proportion of immune cells infiltrating AS, they can still affect the progression of AS by accelerating the formation of apoptotic and necrotic cores in plaques and interfering with the stability of AS plaques through perforin and granzyme. Nevertheless, many specific functions and mechanisms by which NK cells participate in the AS process are still unclear.

#### 4.2.3. DCs

DCs in AS plaques are also a source of foam cells involved in early plaque formation [110], and their number is significantly greater than that in the normal arterial wall, especially in lipid stripes and fibrous plaques. Dendritic cells located in the intima of blood vessels are activated at the early stage of AS. During the process of plaque formation, new DCs are continuously recruited to the lesion area. Factors that damage the endothelium can enhance the adhesion and migration ability of DCs, and oxLDL stimulates ECs to produce GM-CSF, which can increase the adhesion and migration of DCs, thus regulating the number of DCs in plaques [111] (Figure 3). In the advanced plaque of AS, DCs may drive inflammation and disease progression through antigen presentation [112] and/or the production of inflammatory cytokines [113]. Currently, the function of pDCs and their role in AS inflammation are being widely studied. In humans, IFN-α secreted by pDCs leads to the activation of classical dendritic cells (cDCs) and the secretion of proinflammatory factors. IFN-α can also induce effector T cells to increase the levels of IFN-γ and TNF-related apoptosis-inducing ligand (TRAIL) and then kill vSMCs in AS through a TRAIL-DR5-dependent apoptosis mechanism, which may lead to plaque instability and increase the risk of acute coronary syndrome [114]. In an ApoE-deficient mouse model, it was also found that pDC depletion reduced the accumulation of macrophages in plaques and the activation of T cells and reduced the production of proinflammatory cytokines (IL-12, IFN-γ) and chemokines (CXCL1, CXCL10), which had a protective effect on AS [115]. Studies have shown that CCL17 expression can limit the expansion of T cells and accelerate the rupture of plaques. CCL17^+^ DCs play a role in inhibiting Treg cells in AS, and DCs play a central role in the balance of Treg cells in AS lesions [113]. In summary, in different stages of AS, the number of infiltrating DCs is different, which affects the stability and integrity of plaques through various mechanisms, which easily leading to plaque shedding, thrombosis and blood vessel blockage.

#### 4.2.4. Neutrophils

Drechsler and others reported that neutrophils infiltrated mainly into the vascular endothelium and intima in the early stage of AS lesions. With the development of plaque, neutrophils exist in different parts of the plaque, but many neutrophils gather in the lipid core. The size of the AS lesion is positively correlated with the circulating neutrophil count, and the intimal lesion decreases after neutrophil reduction [116]. In the early stage of AS development, injured endothelial cells in plaques bind with neutrophils expressing Mac-1 by producing P-selectin and E-selectin to mediate neutrophil adhesion and infiltration [117]. Myeloperoxidase (MPO), MMP1/MMP2, adhesion molecules and nitric oxide released by activated neutrophil degranulation can cause vascular remodelling and calcification, induce local tissue damage and plaque expansion [118], and secrete a variety of proteases to degrade proangiogenic factors, which hinder angiogenesis and maturation [119]. Neutrophils can also drive Th17 cells and pDCs to release the cytokines IL-1β and TNF-α through neutrophil extracellular traps (NETs), which further promote the inflammatory response in AS [120].

In addition, neutrophils indirectly affect the progression of AS by regulating other immune cells. For example, the matrix metalloproteinase ADAM1 released by neutrophils can cleave CD36, prevent macrophages from phagocytizing apoptotic neutrophils, and promote the formation of necrotic nuclei [121] (Figure 3). Neutropenia can significantly reduce the number of monocytes and macrophages in arterial plaques, which indicates that neutrophils are an important medium for early monocyte recruitment. The relationship between the active substances released by neutrophils and the progression of AS may play a role in the early mediation of monocyte adhesion and aggregation in AS lesions [116]. After neutrophils adhere to ECs, they release soluble components, such as protease 3 (PR3), cytarabine and geraniol, which are beneficial for promoting the recruitment of monocytes and enhancing their adhesion ability [122,123]. Neutrophils in AS are overactivated under various stimuli, and mainly participate in the pathological process of AS by increasing their number, activating degranulation, forming NETs, secreting cytokines and regulating other immune cells and promoting the formation, development, rupture and thrombosis of AS plaques. The inhibition of neutrophil overactivation may provide a new method for the clinical treatment of AS, but the exact mechanism underlying neutrophil involvement in AS injury needs in-depth and comprehensive study.

## 5. Discussion

### 5.1. The diversity of vascular immune regulation and limitations of research in this field

Innate immune cells tend to differentiate into specific phenotypes exercising their respective functions in two different states, health and disease. Innate immune cells at homeostasis are primarily active in processes related to embryonic development and tissue repair. They respond to physiological vascular development by differentiating into pro-angiogenic phenotypes or secreting appropriate amounts of vascular growth factors, acting as ‘coordinators’ and ‘watchdogs’ in tissue homeostasis. When stimulated by diseases, Innate immune cells differentiate into specific disease-related phenotypes, activate strong paracrine ability and cooperate with other immune cells and vascular wall cells to play a regulatory role, playing the role of ‘combatants’ and ‘propagandists’.

In tumours, the superior angiogenesis-promoting ability of immune cells causes ECs to become overactivated and proliferate, which often leads to an imbalance in vascular development, disorder, distortion and leakage of blood vessels and the formation of a vascular network with uneven external and insufficient internal structure, which easily promotes disease metastasis, invasion and a tendency towards bleeding. In other cardiovascular diseases, immune cells that adhere to and infiltrate the vascular wall can also fully act on ECs in the intima and vSMCs in the media and secrete inflammatory factors, enzymes and toxins that damage the vascular wall structure to regulate the stability and permeability of the internal structure of blood vessels, leading to stenosis or the occlusion of blood vessels. In fact, the regulation of angiogenesis by immune cells is not singular but diverse, comprising: (1) Diversity of regulation levels. In the same state, immune cells can play both angiogenic and antiangiogenic roles, and the balance of their angiogenic activities may be affected by the microenvironment, however the specific mechanism is still unclear. (2) Diversity of regulatory mechanisms. Inherent immune cells, especially macrophages, not only play a regulatory role in the early and mature stages of vascular formation but also participate in vascular regulation through paracrine effects, physical traction and direct guidance of vascular buds. Other innate immune cells can also coordinate vascular development in diseased blood vessels with their corresponding phenotypes and multiple mechanisms.

However, inherent immune regulation still has the following limitations: (1) The regulatory mechanisms of innate immune cells tend to be the same. Although most innate immune cells play a direct and indirect roles in vascular diseases, the regulation of angiogenesis is still mainly paracrine regulation, and there is no significant difference between these types of immune cells. Therefore, the unique regulatory mechanism of different types of innate immune cells on blood vessels remains to be studied. (2) The mutual regulatory mechanism between innate immune cells and vascular wall cells is unclear. In the process of immune cells playing a regulatory role, the relationships between cells are not independent, and the interactions between immune cells and vascular wall cells constitute a complex regulatory network. For example, whether TNF-α secreted by M1-like macrophages in the tumour microenvironment can promote angiogenesis depends on the amount of TNF-α secreted and whether pericytes are present. In the absence of pericytes, the higher the concentration of TNF-α is, the stronger the antiangiogenic effect. The presence of pericytes can diminish the antiangiogenic effect caused by a high concentrations of TNF-α [124]. Different degrees of infiltration of innate immune cells can be observed in histopathological samples from patients with obstructive vascular disease and proliferative vascular disease. However, the degree and mechanism of interaction between different types of immune cells and peripheral mural cells are still unclear, and the regulatory effect of mural cells on immune cells may become an important driving factor for the balance between antiangiogenic effects and the promotion of angiogenesis in immune cells. (3) Research on the regulation of blood vessels by the metabolism of innate immune cells is relatively scarce. Currently, most of the research in this field still focuses on cellular secretion products such as vascular growth factors and cytokines released by immune cells, while research on the regulation of angiogenesis by metabolic changes in cells is relatively scarce. However, future development in this field is bound to reveal new targets for immunotherapy of vascular-related diseases and tumours.

### 5.2. Clinical treatment strategies involving vascular immune regulation

Currently, the anti-abnormal angiogenesis drugs widely used in the clinic are mainly VEGF monoclonal antibodies and vascular endothelial growth factor receptor tyrosine kinase inhibitors (VEGFR-TKIs). Although these drugs have definite curative effects and broad application prospects and can be combined with traditional chemotherapy drugs, but their toxicity and side effects cannot be underestimated. Common adverse reactions include bleeding, hypertension, proteinuria, abdominal pain and diarrhoea, hand-foot syndrome, cardiotoxicity and so on [125–128]. For example, bevacizumab is the first antitumour angiogenesis drug approved by the Food and Drug Administration (FDA) in the United States. It can bind to VEGF and block its biological activity, and has been widely used to treat colorectal cancer, breast cancer, lung cancer and other tumors [129]. However, during bevacizumab treatment, some patients will suffer from gastrointestinal perforation, bleeding, proteinuria and hypertension [130]. The tyrosine kinase inhibitor (TKI) sorafenib and the tyrosine kinase receptor inhibitor axitinib also have similar adverse effects in the treatment [131,132]. In general, anti-VEGF drugs combined with radiotherapy [133] or chemotherapy [134] can improve the efficacy of cancer treatment and the patient survival rate. In recent years, many frontier scientific studies have gradually shifted their focus from traditional therapy to immunotherapy. Several clinical studies have shown that bevacizumab combined with immunotherapy for the treatment of NSCLC, hepatocellular carcinoma and renal cell carcinoma improves progression-free survival (PFS) and overall survival (OS) after combined therapy compared with single therapy [135]. Nevertheless, each therapy in combination therapy has complex biological effects, which may increase the risk of toxicity if it is not properly combined. For example, a clinical trial on advanced renal cancer revealed that pembrolizumab (PD-L1 monoclonal antibody) combined with axitinib leads to greater risks of proteinuria and is more prone to renal damage [136].

In fact, the number of drugs in research related to immunotherapy has continued to increase, among which CAR-T-cell therapy ranks first. With the discovery of new targets and mechanisms, CAR-NK [137] and CAR-M [138] therapies with NK cells and macrophages as effector cells are increasingly included in the research. Traditional AS therapy still focuses on cholesterol-lowering drugs and a low-fat diet, but some researchers have shifted their direction to immune-related therapy and developed nanodrugs to reshape the atherosclerosis immune microenvironment (AIM). The drug targets immune cells in AS and inhibits the infiltration of immune cells and reduces the production of inflammatory factors by macrophages by blocking the interaction between macrophages and CD8^+^ T/NKT cells, thus providing a feasible strategy for AS immunotherapy [139]. In the process of angiogenesis, innate immune cells play a variety of regulatory roles, and macrophages can initiate the formation of new blood vessels and the maturation of blood vessel networks by secreting angiogenic factors and matrix remodelling proteases, guiding blood vessel buds and pruning blood vessel networks. In the advanced tumour stage, M2-like macrophages produce high-level proangiogenic signals to regulate the ‘angiogenesis switch’ and thus promote tumour progression [140]. Researchers have reported that targeted macrophage therapy combined with antiangiogenic drugs can play a synergistic role in tumours. In a mouse model of breast cancer, a fasting-mimicking diet (FMD) can inhibit the infiltration and activity of M2-like TAMs, which combined with apatinib can produce synergistic antitumour activity [141]. In addition, the latest research found that the combination of anti-glutamine metabolic inhibitors and antiangiogenic drugs in HER2-positive gastric cancer provides a new direction for reversing the drug resistance of trastuzumab, which highlights the synergistic effect of targeted macrophage metabolism therapy and antiangiogenic therapy [142]. Therefore, (1) TAM clearance therapy including CSF1R-blocking antibodies and inhibitors, small-molecule inhibitors and selective clearance of M2-like macrophages [143]. (2) TAM recruitment therapy including CCL2-CCR2 inhibitors [144] and CXCR4 antagonists [145]. (3) M2-like TAM phenotypic conversion therapy including CSF1R inhibitors, corosolic acid, omeprazole, Gpr132 inhibitors, MEK/STAT3 inhibitors, FMD, and antibodies against IL-4 [146] will provide new combination strategies for antiangiogenic therapy.

Innate immune cells, such as DCs, can secrete antiangiogenic factors in specific environments, such as inflammation and tumours. Exploring the synergistic effects of antiangiogenic effects on immune cells and antiangiogenic drugs will provide a new direction for antiangiogenic strategies. Additional studies of immunotherapy in combination with small-molecule anti-angiogenic drugs are currently underway. However, both immunomodulation and angiogenesis are essential processes for maintaining homeostasis in various organs throughout the body. Systemic administration usually cannot avoid adverse reactions and off-target effects. Therefore, it is important to improve the binding ability of small-molecular targets to their receptors or to improve the effective aggregation of drugs in target tissues through immune cells [147]. Recent studies have found that delivery of siRNA by constructing target cell-derived hybrid exosome vesicle (HEV) enables tissue-specific therapy. This novel approach targets the eye to treat dry eye disease [148]. This suggests that tissue-specific drug delivery strategies should not be overlooked. Therefore, optimizing drug targeting strategies and developing tissue-specific precision therapies could not only provide direction for reducing the side effects of antiangiogenic therapies, but also provide ideas for treating diseases with vascular abnormalities at a holistic level. It is worth noting that in the era of combination drugs, combination therapy remains a ‘double-edged sword’. How to control the dosage, time and the order of use of drugs to maximize the synergistic benefits remains a challenge. In the future, we should conduct more detailed and comprehensive research on the mechanism of joint action, optimize the joint scheme, and provide safer and more personalized treatment for patients.

## Data Availability

Data sharing is not applicable to this article as no new data were created or analysed in this review.
